# Modifiable risk factors in adults with and without prior cardiovascular disease: findings from the Indonesian National Basic Health Research

**DOI:** 10.1186/s12889-022-13104-0

**Published:** 2022-04-05

**Authors:** Dian Sidik Arsyad, Jan Westerink, Maarten J. Cramer, Jumriani Ansar, Wahiduddin, Frank L. J. Visseren, Pieter A. Doevendans, Ansariadi

**Affiliations:** 1 Department of Cardiology, Division of Heart and Lungs, University Medical Centre Utrecht, University of Utrecht, 3584 CT Utrecht, The Netherlands; 2 Department of Epidemiology, Faculty of Public Health, Hasanuddin University, Makassar, Indonesia; 3 Department of Vascular Medicine, University Medical Center Utrecht, Utrecht, The Netherlands; 4 Netherlands Heart Institute Utrecht, Utrecht, The Netherlands

**Keywords:** Cardiovascular diseases, Epidemiology, Lifestyle, Prevalence, Prevention, Risk factors

## Abstract

**Backgrounds:**

The majority of risk factors for cardiovascular diseases (CVDs) are modifiable. Continuous monitoring and control of these factors could significantly reduce the risk of CVDs-related morbidity and mortality. This study estimated the prevalence of modifiable risk factors in Indonesia and its co-occurence of multiple risk factors stratified by prior CVDs diagnosis status and sex.

**Methods:**

Adult participants (> 15 years, *N* = 36,329, 57% women) with median age of 40 years were selected from a nationwide Indonesian cross-sectional study called Basic Health Research or Riset Kesehatan Dasar (Riskesdas) conducted in 2018. Thirteen risk factors were identified from the study, including smoking, a high-risk diet, inadequate fruit and vegetable consumption, a low physical activity level, the presence of mental-emotional disorders, obesity, a high waist circumference (WC), a high waist-to-height ratio (WtHR), hypertension, diabetes, a high total cholesterol level, a high low-density lipoprotein (LDL) cholesterol level, and a low high-density lipoprotein (HDL) cholesterol level. Age-adjusted prevalence ratios stratified by CVDs status and sex were calculated using Poisson regression with the robust covariance estimator.

**Results:**

CVDs were found in 3% of the study population. Risk factor prevalence in the overall population ranged from 5.7 to 96.5% for diabetes and inadequate fruit and vegetable consumption respectively. Smoking, a high-risk food diet, and a low HDL cholesterol level were more prevalent in men, whereas a low physical activity level, the presence of mental-emotional disorders, obesity, a high WC, a high WtHR, hypertension, diabetes, a high total cholesterol level, and a high LDL cholesterol level were more prevalent in women. Approximately 22% of men and 18% of women had at least 4 risk factors, and these proportions were higher in participants with prior CVDs diagnosis.

**Conclusions:**

There is a high prevalence of modifiable risk factors in the Indonesian adult population. Sex, age, and the presence of CVD are major determinants of the variations in risk factors. The presence of multiple risk factors, which are often inter-related, requires a comprehensive approach through health promotion, lifestyle modification and patient education.

## Background

Cardiovascular diseases (CVDs) is a major cause of morbidity and mortality worldwide [1]⁠. Although the risk factors underlying CVDs are well known and, for the most part, preventable, the prevalence is still increasing [2]⁠. Prevention guidelines recommend lowering the risk by influencing modifiable risk factors to reduce morbidity and mortality [3, 4]⁠. The prevalence of CVDs is also increasing in Southeast Asia, including Indonesia, increasing from 4.8 to 5.3% between 2014 and 2019 [5]⁠. In Indonesia, the prevalence of CVDs varies according to geographic and demographic characteristics, including between men and women, presumably due to differences in exposure to risk factors [6]⁠. Furthermore, there are significant gender differences in the presence risk factors and the occurrence of CVDs. Although men have a higher risk of coronary heart disease (CHD), women have an equal or even higher risk for stroke [7, 8]⁠.

Considerable evidence has shown that many cardiovascular risk factors are inter-correlated, and exposure to multiple factors significantly increases the risk of CVDs incidence [9]⁠. For example, hypertension, type 2 diabetes, hyperlipidemia, and obesity are closely related to unhealthy behavior and lifestyle such as smoking, high risk food diet, low physical activity,and stress [10]⁠.

Limited information is available regarding the variation, burden, and co-occurrence of cardiovascular risk factors in the Indonesian population. Reliable epidemiological data of modifiable risk factors prevalence and variation among the Indonesian population are fundamental in shaping the strategic approach to decreasing the risk of CVDs. Therefore, the aims of this study are (1) to provide up-to-date prevalence of modifiable risk factors, (2) to describe the variations in risk factor distribution in the populations with a prior CVDs diagnosis (secondary prevention population) and without a CVDs diagnosis (primary prevention population), and sex. In addition, (3) to describe the extent of co-occurrence of multiple risk factors in the Indonesian adult population.

## Methods

### Study design and data source

The current study is based on the National Basic Health survey, or Riset Kesehatan Dasar (Riskesdas). This five-year cross-sectional study began in 2007 and was conducted throughout the country by the Ministry of Health, the Republic of Indonesia. The principal aim of the Riskesdas was to provide information to the Indonesian government concerning population health status and associated risk factors [6]⁠. Detailed information about the study design, data management, and methods of the Riskesdas has been described previously [11]⁠. In brief, the target sample was 300.000 households from 30.000 pre-listed census blocks (CBs) provided by the Indonesian Central Bureau of Statistics. These CBs are distributed among 34 provinces in 514 districts/cities throughout Indonesia. Recruited and well-trained enumerators visited every randomly selected household and invited all its members to participate in a questionnaire-based interview. Furthermore, height, weight, waist circumference (WC), and blood pressure measurements were obtained.

Adult household members (15 years and older) from 2500 CBs in 26 provinces were sub-sampled to represent national-level prevalence estimates for blood examinations (fasting [FBG] or random blood glucose [RBG] and cholesterol profiles). The response rate for blood examination was 77.7%, and in the present study, we included only participants for whom blood samples were available (*N* = 36,329).

### Data collection

The presence of CVDs, smoking, food consumption, physical activity, and mental-emotional state were self-reported. Body mass index (BMI), WC, waist-to-height ratio (WtHR), and blood pressure (BP) were measured by the surveyor. Blood samples were collected at the time of the interview to detect FBG or RBG and total cholesterol, low-density lipoprotein (LDL) cholesterol, and high-density lipoprotein (HDL) cholesterol levels.

We categorized participants as smokers if they currently smoked (daily or occasionally). High-risk food was defined as sweet food and drinks, salty food, oily food, grilled food, preserved food, seasonings, soft drinks, energy drinks, and instant food/noodles. A “high-risk diet” was defined as consuming of three or more types of high-risk food at least once a day. Participants’ fruit and vegetable consumption was categorized as “inadequate” if intake was less than five portions/day according to the World Health Organization (WHO) standard [12]⁠. Physical activity was defined based on the WHO Global Physical Activity Questionnaire (GPAQ) and categorized as “low” if participants did not meet the WHO standard for adequate physical activity [13]⁠. Mental and emotional disorders were measured using the WHO 20-item self-reporting questionnaire (SRQ-20) [14]⁠, which was previously validated for the Riskesdas. We defined the presence of mental and emotional disorders as a score of 6 or above as it is already validated in the other study in Indonesia (with positive predictive value 60%, negative predictive value 92%) [15]⁠, and was used in the Riskesdas national report.

Participants’ anthropometric indices, namely, their weight, height, and WC, were measured using appropriate measuring tools. A digital weight scale with a precision of 100 g was used to measure weight; height was measured using a portable stadiometer with a precision of 0.1 cm, and WC was measured using a measuring tape with a precision of 0.1 cm at the midline between the inferior margin of the ribs and the superior border of the iliac crest. Participants with BMI ≥25 kg/m2 were considered “obese” [16]⁠, and a WC > 90 cm in men and > 80 cm in women was considered “high risk” [17]⁠. In addition, the WtHR was also calculated, and a score > 0.5 was considered “high risk” [18]⁠.

BP was measured with a digital sphygmomanometer at least two times on the upper left arm while the patient was in a relaxed condition, and average systolic and diastolic BP was calculated to represent the participant’s BP. An average systolic BP of ≥ 140 mmHg and or an average diastolic BP of ≥ 90 mmHg, self-reported hypertension diagnosed by a physician, or the use of a BP-lowering medication was defined as “hypertension” [19]⁠. Furthermore, for participants with hyperglycemia symptoms and RBG level ≥ 200 mg/dL or FBG level > 125 mg/dL, a self-reported diagnosis of diabetes or use of anti-diabetic medication was defined as “diabetes” [20]⁠. FBG and RBG data were collected using a point-of-care test (POCT) glucometer. Lipid profiles (total, LDL, and HDL cholesterol levels) were measured in mg/dL. Blood samples collected from participants were sent to the National Laboratory in Jakarta to analyze lipid profiles using enzymatic assays, and total cholesterol ≥ 200 mg/dL, LDL cholesterol ≥ 160 mg/dL, and HDL cholesterol < 40 mg/dL were defined as “high risk” [21]⁠.

The Riskesdas study was approved by the Ethical Committee of Health Research, NIHRD, Ministry of Health, the Republic of Indonesia, with the reference number LB.02.01/2/KE.267/2017. Informed consent was obtained from all participants or their legal guardians (for participants aged 15 years old) who participated in the study.

### Statistical analysis

We conducted descriptive analyses to estimate the prevalence of CVDs (coronary heart disease and stroke) and its modifiable risk factors. Due to the complex survey methodology, adjustment with a sampling weight to address unequal sampling probabilities related to non-responses and sampling design was performed. Age-adjusted prevalence ratios by CVDs status (no prior and a prior CVDs diagnosis) and sex were calculated using Poisson regression with the robust covariance estimator [22]⁠. Categorical variables are expressed as percentages, and chi-square tests were performed to compare the proportions of risk factors between groups. Independent t-tests or Mann–Whitney tests were used for continuous variables to compare mean distributions between two groups.

Eight modifiable risk factors, namely, smoking, a high-risk diet, a low physical activity level, the presence of mental-emotional disorders, a high WtHR, hypertension, a high blood glucose level or diabetes, and a high LDL cholesterol level, were included in the calculation of the participant’s cumulative number of risk factor presented. We excluded inadequate fruit and vegetable consumption because of the high similarities between sex and age groups (homogeneous). BMI and WC were excluded because of their collinearity with WtHR in predicting obesity risk and thus the risk for CVDs. Furthermore, we included LDL cholesterol levels and excluded total and HDL cholesterol levels in the cumulative number of risk factor calculations. The analysis was performed using SPSS version 27 (IBM Corp. Armonk, NY).

## Results

### Study population characteristics

**Fig. 1:**
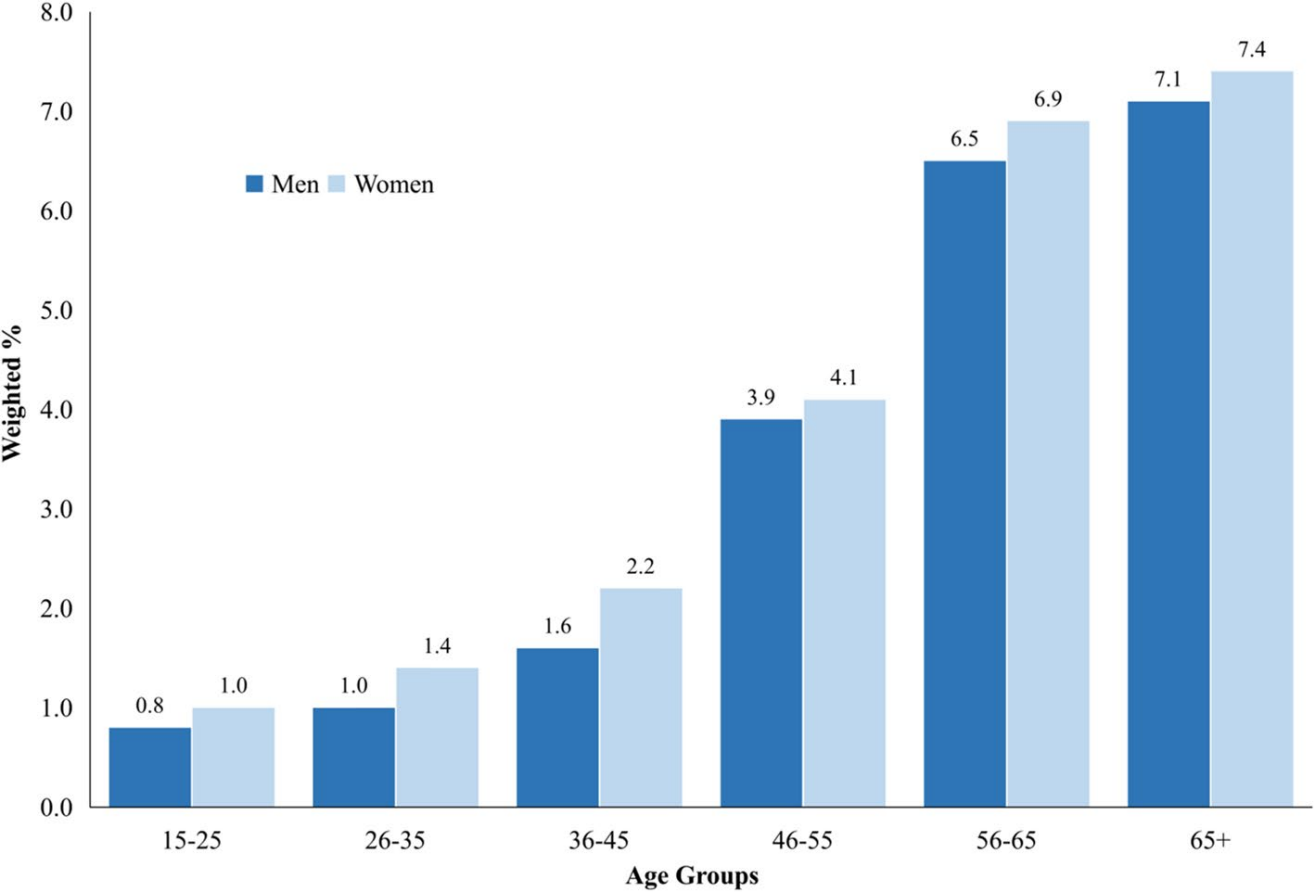
Prevalence of Cardiovascular Diseases (CHD and Stroke) By Sex and Age Group. Dark blue color represents the prevalence of risk factors in men, while light blue color represents the prevalence of risk factors among women. Horizontal axis is age groups in 10-year intervals. The prevalence presented in weighted percentage (%)

A total of 36,329 participants were included in our analysis, and the study population characteristics are presented in Table 1. The median ages of participants with and without a prior CVDs diagnosis were 54 and 40, respectively, and more than half were women in both groups. The weighted prevalence of CHD and stroke were 2.1% (95% CI 1.9–2.3) and 1.0% (95% CI 0.9–1.1), respectively, and the prevalence of combined CHD and stroke was 3.0% (95% CI 2.8–3.2). The prevalence increased with age in both men and women. The CVDs prevalence by 10-year age intervals and sex is presented in Fig. 1.

**Table 1:** Characteristics of the participants by prior diagnosis of CVDs status

Characteristics	Prior CVDs Diagnosis (% weighted)			p
	No (***n*** = 35,129)	Yes (***n*** = 1200)	Total (***n*** = 36,329)	
**Age (years)**; median (IQR)	40 (24)	54 (19)	40 (23)	< 0.001
**Sex**				
Men	43.3	41.4	43.2	0.323
Women	56.7	58.6	56.8	
**Location**				
Urban	63.8	69.3	63.9	0.001
Rural	36.2	30.7	36.1	
**Marital status**				
Not Married	16.5	6.1	16.2	< 0.001
Married	74.1	74.9	74.2	
Divorced	2.1	1.7	2.1	
Widowed	7.2	17.3	7.5	
**Education**				
Not / Never School	5.9	8.9	6.0	< 0.001
Not Finished Primary	13	18.1	13.1	
Primary School	29.3	30.1	29.3	
Junior Highschool	20.9	16.9	20.8	
Senior Highschool	25.1	19.9	24.9	
Undergraduate School	2.3	2.3	2.3	
Finished Higher Education	3.6	3.7	3.6	
**Occupation**				
Not Working	35.5	49.0	35.9	< 0.001
Schooling	6.1	2.3	6.0	
Government Employee	1.3	1.6	1.4	
Private Employee	8.9	4.7	8.8	
Enterpreneur/Enterpriser	14.8	15.5	14.8	
Farmer	15.9	13.5	15.8	
Fisherman	0.3	0.1	0.3	
Daily labour	12.0	6.6	11.8	
Other	5.2	6.7	5.2	
**Measurements;** mean (SD)				
BMI score	23.9 (4.8)	24.6 (5.1)	23.9 (4.8)	< 0.001
WC (cm)	79.9 (12.2)	84 (13.6)	80 (12.3)	< 0.001
WHtR	0.52 (0.08)	0.54 (0.09)	0.52 (0.1)	< 0.001
Systolic (mmHg)	131.2 (23.7)	145 (29.6)	131.5 (24.2)	< 0.001
Diastolic (mmHg)	83.8 (12.6)	88 (15.4)	83.9 (12.8)	< 0.001
FBG (mg/dL) (n:10083)	102.1 (31.9)	111.1 (45.4)	102.4 (32.5)	< 0.001
RBG (mg/dL) (n:26246)	112.1 (45.2)	120.5 (56.6)	112.3 (45.6)	0.017
Total-Cholesterol (mg/dL)	181.3 (39.9)	191.7 (44.6)	181.6 (40.1)	< 0.001
LDL-Cholesterol (mg/dL)	122.0 (33.9)	129.4 (37.3)	122.3 (34.1)	< 0.001
HDL-Cholesterol (mg/dL)	48.1 (11.3)	47.6 (11.8)	48.1 (11).3	0.185

Age is presented in median (IQR), participant characteristics (sex, location, marital status, and education) are presented in weighted percentage (%), measurements (BMI, blood pressures, blood glucose, and lipid profile) presented with a mean (with standard deviation); significant values were derived from t-test or Mann-Whitney tests for continuous variables, and chi-square test for categorical variables; *p*-values less than 0.05 were considered statistically significant

*CVDs* Cardiovascular disease, *CI* confidence interval, *IQR* interquartile range, *SD* standard deviation, *BMI* body mass index, *WC* waist circumference, *WHtR* waist to height ratio, *FBG* fasting blood glucose, *RBG* random blood glucose, *LDL* low-density lipoprotein, *HDL* high-density lipoprotein

### Prevalence of modifiable risk factors by CVDs status

**Fig. 2:**
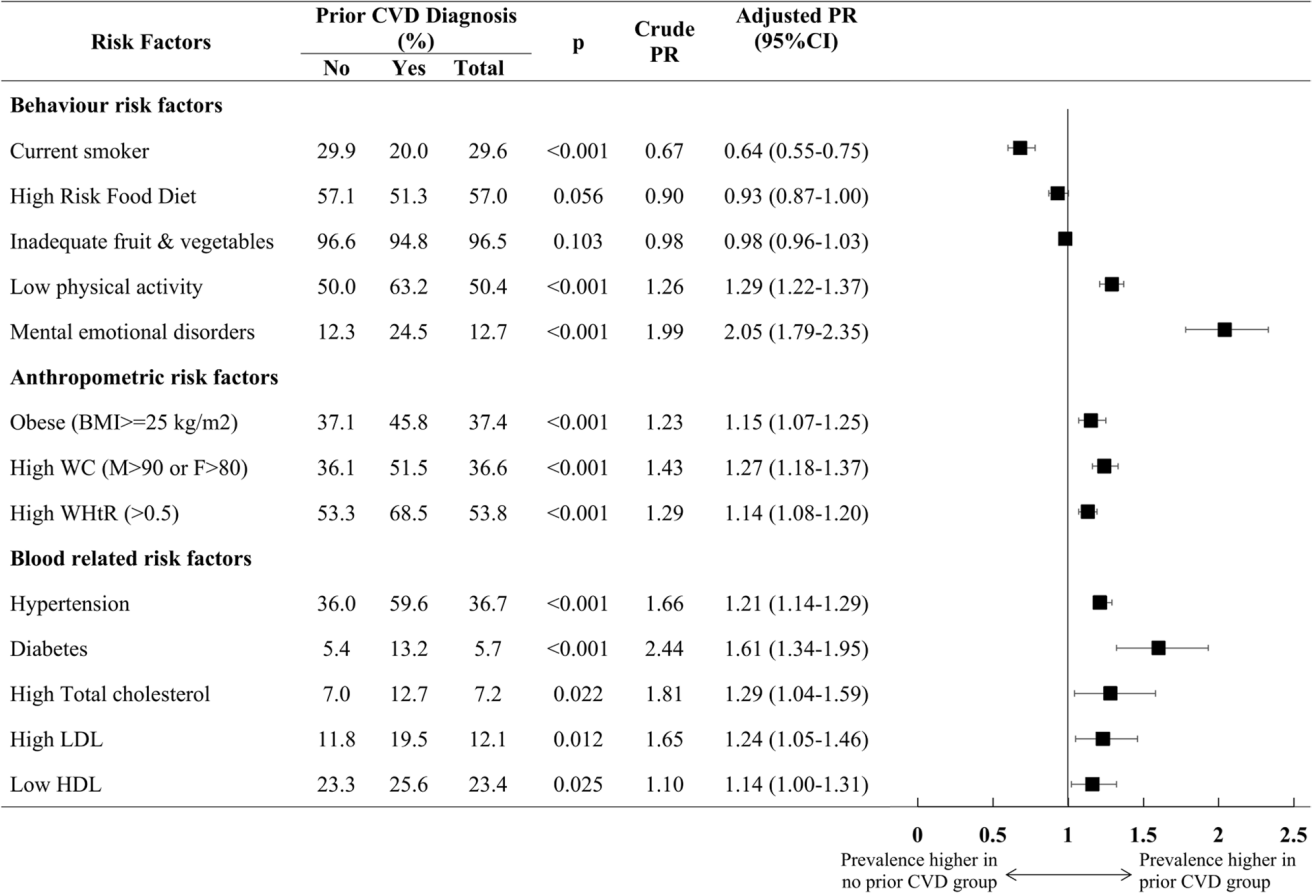
Prevalence of modifiable risk factors by prior CVDs diagnosis. Modifiable risk factors were differentiated into three categories (behavior risk factors, anthropometric risk factors, and blood-related risk factors); estimated prevalences for with and without prior CVDs diagnosis are presented in percentage (%), Poisson regression with robust covariance estimator was performed to calculate prevalence ratio adjusted for age and sex with 95% CI and statistical significance of the prevalence ratios. *P*-values less than 0.05 were considered statistically significant. CVDs:cardiovascular disease; CI:convidence interval; PR:prevalence ratio; BMI:body mass index; WC:waist circumference; WHtR:waist to height ratio; LDL: low-density lipoprotein cholesterol; HDL:high-density lipoprotein cholesterol

The prevalence of cardiovascular risk factors based on a history of CVDs diagnosis is presented in Fig. 2. The prevalence in the overall population ranged from 5.7% for diabetes to 96.5% for low fruit and vegetable consumption. Participants with CVDs had significantly higher prevalence for most of the risk factors, except for smoking, than those with no prior CVDs (PR: 0.64, 95% CI 0.55–0.75, *p* < 0.001); there was no significant difference in the prevalence of a high-risk diet (PR: 0.93, 95%CI 0.87–1.00, *p* = 0.056) and inadequate fruit and vegetable consumption (PR:0.98, 95%CI 0.96–1.03, *p* = 0.103) between the groups. The highest prevalence difference between the groups was for mental-emotional disorders, for which participants with prior CVDs had a significantly higher prevalence (PR: 2.05, 95% CI 1.79–2.35, *p* < 0.001).

### Prevalence of modifiable risk factors by sex

**Fig. 3:**
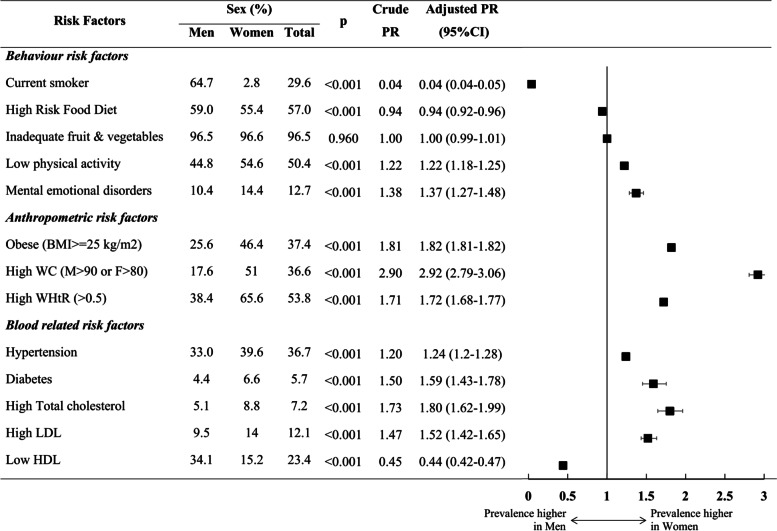
Prevalence of modifiable risk factors by sex. Modifiable risk factors were differentiated into three categories (behavior risk factors, anthropometric risk factors, and blood-related risk factors); estimated prevalences for men and women are presented in percentage (%), Poisson regression with robust covariance estimator was performed to calculate prevalence ratio adjusted for age and prior CVDs status with 95% CI and statistical significance of the prevalence ratios. *P*-values less than 0.05 were considered statistically significant. CVDs:cardiovascular disease; CI:convidence interval; PR:prevalence ratio; BMI:body mass index; WC:waist circumference; WHtR:waist to height ratio; LDL: low-density lipoprotein cholesterol; HDL:high-density lipoprotein cholesterol

The prevalence of a majority of risk factors, including a low physical activity level, the presence of mental-emotional disorders, obesity, a high WC, a high WtHR, hypertension, diabetes, a high total cholesterol level, and a high LDL cholesterol level, were significantly higher in women than in men. On the other hand, smoking, a high-risk diet and a low HDL cholesterol level were more common among men. Furthermore there was a notable difference in smoking prevalence when comparing women and men (PR: 0.04, 95% CI 0.03–0.05, *p* < 0.001). A high WC was more common in women than in men (PR: 2.92, 95% CI 2.79–3.01, *p* < 0.001). There were no significant differences in fruit and vegetable consumption between the sexes (PR:1.00, 95%CI 0.99–1.01, *p* = 0.960). Information regarding the prevalence ratios of modifiable risk factors by sex is shown in Fig. 3.

### Multiple CVDs risk factor co-occurrences

**Fig. 4:**
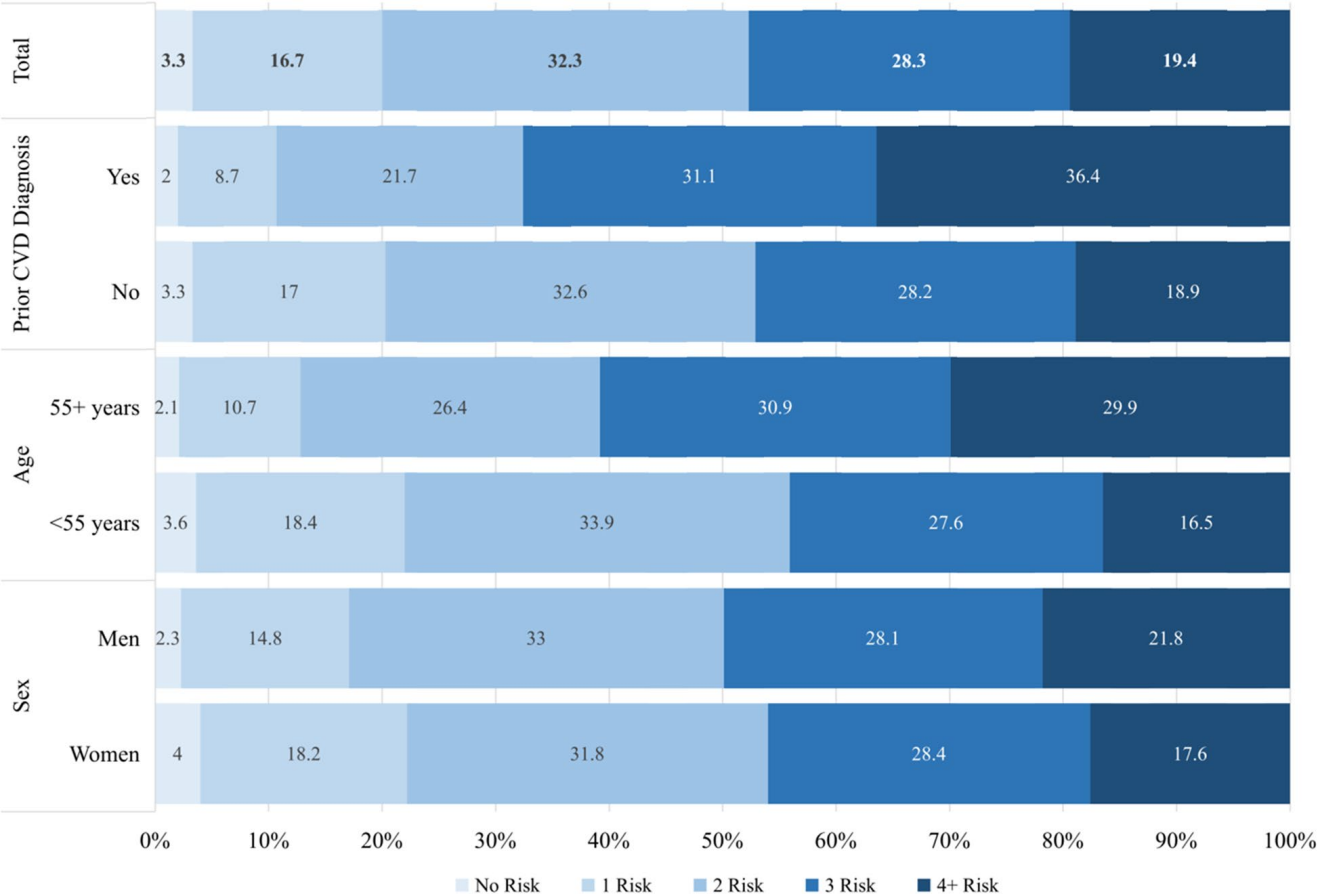
Cumulative number of risk factors among adult by cardiovascular disease (CVDs) diagnosis, sex and age. The cumulative number of CVDs risk factors selected for the calculation are current smokers, high-risk food diets, low physical activity, mental-emotional disorder, high waist to height ratio (WHtR), high blood pressure or hypertension, diabetes, and high low-density lipoprotein (LDL). The horizontal axis represents the percentage of participants in the study, while the vertical axis represents a group of participants by sex (men and women) and by age groups(< 55 years and ≥ 55 years); graduated colors are representing the number of experienced risk factors which differentiated into 5 category

Co-occurrence of risk factor in participants was presented with the cumulative number of CVDs risk factor in Fig. 4. Out of eight risk factors included in the calculation, the median number of multiple risk factors co-occurrence for participants with and without prior CVDs were 3 and 2 risk factors, respectively. Only 3.3% of the overall participants had no modifiable risk factors, while 18.9% of the participants without CVDs and 36.4% of the participants with CVDs had at least 4 modifiable risk factors. The percentage distribution of co-occurrence of multiple risk factors also differed between sex and age groups.

## Discussion

The majority of the adult population in Indonesia had modifiable cardiovascular risk factors, with 80% having two or more risk factors, independent of the presence of prior CVDs. There were substantial differences in the modifiable risk factor prevalence between men and women. These results highlight the importance of improvements in cardiovascular risk management in patients with and without CVDs. In addition, a differential approach to risk factor management based on sex seems warranted. The overall prevalence of CVDs in the current study was lower than that in the Global Burden of Disease (GBD) Study 2019 (5.3%) [5]⁠, this was most likely due to self-reporting and only CHD and stroke were included in the current study.

### Modifiable risk factors in the Indonesian adult population

Tobacco smoking contributes to almost 50% of cardiovascular-related deaths in the Southeast Asian region [23]⁠. In the current study, 29.6% of adults were active smokers, and this proportion was higher than the global average [24]⁠. Althought a study by Yusuf et.al showed that among behavioral risk factors, tobacco smoking was the most strongly associated with the occurrence of CVDs [25]⁠, our study indicated that 20% of participants with a prior CVDs diagnosis were still active smokers, The prevalence of smoking was extremely high in men (64.7% vs. 2.8% in women) and there was no significant change from 2013 in both sexes [26]⁠. A study by Marie Ng et.al showed an average of cigarette consumption in Indonesia was 11 sticks per day, which was lower than global average of 18 sticks per day [27]⁠. The distribution of tobacco smoking between men and women in the current study is commonly found in the Southeast-Asian populations but differs from the western population where the number of smoking women almost as high as in men [24]⁠. Despite of national efforts to reduce tobacco consumption, smoking remains a major risk factor for CVDs in Indonesia.

Many studies have shown that an unhealthy diet (high-risk food intake or inadequate fruit and vegetable consumption) is associated with an increased risk of cardiovascular events [28]⁠. We found that in Indonesia, a high proportion of participants consumed high-risk food and had inadequate fruit and vegetable consumption, independent of the presence of CVDs. This is in line with the result of other studies that found the majority of the population still consumes less fruit and vegetables than recommended by the standard [29, 30]⁠.

There is a clear association between adequate physical activity with better cardiovascular health and diabetes in which lifestyle interventions based on physical activity are promoted for both primary and secondary care [31]⁠. However, our analyses demonstrated a significant difference in physical activity level between participants with and without CVDs, approximately 6 out of 10 participants with known CVDs diagnosis had a low physical activity level according to the WHO standard. Although the prevalence of an inadequate physical activity level was lower in participants without CVDs in our study, it was still much higher than other East and Southeast Asian countries [32]⁠. Several factors might influence the physical activity level. In Indonesia, rapid urbanisation, economic development, and technology advancement, particularly transportation creating an environment conducive to sedentary behavior and physical inactivity, might partly explain the situation.

Mental and emotional disorders were more prevalent in participants with a prior CVDs diagnosis and more common among women than men; our finding was in line with those of other studies [33]. The underlying reason for the variation in the presence of mental and emotional disorders between sexes remains unclear. Associations of anxiety, stress, and depressive disorder with an increased risk of CVDs have been shown in healthy and pre-existing CVDs populations [34]⁠. Although not included in our analysis, disease risk perception is an important risk factor worth mentioning. According to studies from Indonesia, people who have a higher disease perception positively correlated with better cardiovascular health behavior and medication adherence for those who have already developed risk factors and have been diagnosed with CVDs [35, 36]⁠. Thus, understanding a person’s perception of a disease is essential for health professionals when planning and delivering prevention and treatment strategies. The high prevalence of mental and emotional disorders among participants in our study, especially in the manifested CVDs population, may suggest that improving knowledge and disease perception, creating awareness, and addressing mental problems among CVDs patients by health care services would be beneficial [37]⁠.

Anthropometric measurements for body fat distribution, including BMI, WC, and WtHR, are good predictors of cardiovascular health and the risk of cardiovascular events [38, 39]⁠. Obese, a high WC, and a high WtHR were common in participants with a prior CVDs diagnosis and consistently high among women. Our findings agree with those of other studies [40–42]⁠. Complex hormonal and metabolic fluctuations in their life course combined with unhealthy lifestyles, including a low physical activity level and an unhealthy diet, are possible explanations for why women have a higher risk of anthropometric indices than men [43]⁠.

The prevalence of major risk factors, namely hypertension, diabetes, and hyperlipidemia, in the current study, were higher than those in the previous Riskesdas in 2013 [26]⁠. Furthermore, excluding a low HDL cholesterol level, we found these risk factors significantly more prevalent in the prior CVDs group and in women. Our findings differed from the global pattern, in which the prevalence of hypertension, diabetes, and cholesterol were higher among men [2]⁠. Two methods of estimating hypertension and diabetes prevalence were used in the Riskesdas study, firstly, estimation of prevalence based on the participants’ self-reporting of physician diagnosis, secondly, with direct blood pressure and blood glucose level measurement during a house visit. A significant prevalence discrepancies between both methods where self-reported assessment shows lower estimates than direct measurements indicate a missed opportunity for diagnosis among this population.

The present study explored the co-occurrence of multiple CVDs risk factors (also known as risk factors clustering) in the Indonesian adult population. Our study demonstrated that the co-occurrence of multiple risk factors is highly prevalent. Approximately 1 out of 5 adults have at least four risk factors of CVDs, even higher in the manifested CVDs group and older group (> 55 years) and slightly higher in men. The risk factors clustering pattern in Indonesia seems comparable to other studies [9, 44, 45]⁠. Risk factors for CVDs are typically clustered in an individual due to the inter-correlation of risk factors with each other. Unhealthy lifestyles and behavior are known to be associated with increased risk of metabolic syndrome, including hypertension, dyslipidemia and diabetes which eventually increases the likelihood of developing CVDs.

In Indonesia, several initiatives have been implemented to reduce the burden of CVDs [46]⁠. Screening for NCD risk factors through the Posbindu, comprehensive training on screening and managing NCD risks for health professionals and health volunteers, implementing tobacco control measures, and strengthening the capacity of the Puskesmas for prevention and control are several examples of nationwide efforts to reduce the disease burden [46]⁠. Nevertheless, as the current study highlights, the prevalence of modifiable risk factors for CVDs remains high. A study conducted by Maharani and Tampubolon [47]⁠ demonstrated that the high prevalence of modifiable risk factors for CVDs in Indonesia is due to unmet needs for care and is strongly correlated with socioeconomic factors. The Indonesian government introduced the National Healthcare Insurance program (*Jaminan Kesehatan Nasional* or JKN) in 2014 to ensure the availability of medication for primary and secondary prevention and adequate access to quality health services, including CVDs care [48]⁠.

Despite numerous efforts to reduce the burden of CVDs and their risk factors, limited analyses of these efforts have considered population heterogeneity [49]⁠. Future research and prevention strategies for CVDs in Southeast Asia, particularly Indonesia, should consider the broad range of risk factors, the heterogeneity of the population, and the co-occurrences of multiple risk factors.

### Strengths and limitations of the study

To the best of our knowledge, this study is the first to investigate the estimated prevalence of modifiable risk factors in participants with and without CVDs using representative Indonesian national-level data. Furthermore, the large sample size provided sufficient power to calculate the prevalence of risk factors to represent national estimates. Several important limitations were identified, including the self-reporting of CVDs diagnosis and excluding peripheral artery disease, potentially leading to an underestimation of the prevalence of CVDs. A self-reported assessment, especially in behavioral risk factors, may introduce recall bias that possibly hinders our findings. As participants tend to overestimate exercise and underestimate food intake, our findings may thus be an underestimation of the already high prevalence of cardiovascular risk factors in this population. Future studies could include specific food and exercise recall forms to limit this bias.

Moreover, the smoking quantity was also not available from our data, thus preventing us from further analyzing the amount of tobacco consumption patterns. There was only a 77.7% response rate among the targeted participants for blood examination; this may have led to sampling bias which could affect the accuracy of the prevalence estimation. Furthermore, the study did not include other known risk factors, such as excessive alcohol consumption, household indoor air pollution, family history of CVDs, ethnicity, or perceived risk and awareness of the disease.

## Conclusions

Independent of the presence of prior CVDs, the majority of the adult population in Indonesia has at least two modifiable cardiovascular risk factors. Furthermore, the prevalence of modifiable risk factors differed significantly between men and women. These findings highlight the importance of addressing the risk factor variation and developing a differential and comprehensive approach to risk factor control based on sex and the co-occurrence of multiple risk factors in primary and secondary prevention populations to reduce the CVDs burden in Indonesia.

## Data Availability

The dataset analyzed for the current study is not publicly available, but the data may be available from the Data Management Laboratory of NIHRD, Ministry of Health Republic of Indonesia on reasonable request with prior officially written permission.

## Abbreviations

CVDs: Cardiovascular Diseases
GBD: Global Burden of Disease
NCD: Non-Communicable Disease
Puskesmas: Pusat Kesehatan Masyarakat (Public Primary Health Care)
Posbindu: Pos Pembinaan Terpadu (Integrated Guidance Posts)
CBs: Census Blocks
CI: Convidence Interval
MI: Myocardial Infarction
WHO: World Health Organization
SRQ: Self Reporting Questionnaire
GPAQ: Global Physical Activity Questionnaire
POCT: Point of Care Test
JKN: Jaminan Kesehatan Nasional (National Health Insurance)
CBS: Census Blocks
PR: Prevalence ratio
BMI: Body mass index
WC: Waist circumference
WHtR: Waist to height ratio
BP: Blood pressures
BG: Blood glucose
FBG: Fasting blood glucose
RBG: Random blood glucose
LDL: Low-density lipoprotein
HDL: High-density lipoprotein

## References

[1] Mensah GA, Roth GA, Fuster V (2019). The global burden of cardiovascular diseases and risk factors: 2020 and beyond. J Am Coll Cardiol.

[2] Roth GA, Mensah GA, Johnson CO, Addolorato G, Ammirati E, Baddour LM (2020). Global burden of cardiovascular diseases and risk factors, 1990–2019. J Am Coll Cardiol.

[3] Visseren FLJ, Mach F, Smulders YM, Carballo D, Koskinas KC, Bäck M (2021). 2021 ESC guidelines on cardiovascular disease prevention in clinical practice. Eur Heart J.

[4] Arnett DK, Blumenthal RS, Albert MA, Buroker AB, Goldberger ZD, Hahn EJ (2019). 2019 ACC/AHA guideline on the primary prevention of cardiovascular disease. J Am Coll Cardiol.

[5] Institute for Health Metrics and Evaluation (IHME) (2019). GBD compare.

[6] National Institute of Health Research and Development (NIHRD) (2019). National Basic Health Research (Riskesdas) Report 2018.

[7] Leening MJG, Ferket BS, Steyerberg EW, Kavousi M, Deckers JW, Nieboer D (2014). Sex differences in lifetime risk and first manifestation of cardiovascular disease: prospective population based cohort study. BMJ..

[8] Manfrini O, Yoon J, van der Schaar M, Kedev S, Vavlukis M, Stankovic G (2020). Sex differences in modifiable risk factors and severity of coronary artery disease. J Am Heart Assoc.

[9] Peters SAE, Wang X, Lam T-H, Kim HC, Ho S, Ninomiya T (2018). Clustering of risk factors and the risk of incident cardiovascular disease in Asian and Caucasian populations: results from the Asia Pacific cohort studies collaboration. BMJ Open.

[10] van Oort S, Beulens JWJ, van Ballegooijen AJ, Grobbee DE, Larsson SC (2020). Association of cardiovascular risk factors and lifestyle behaviors with hypertension. Hypertension..

[11] National Institute of Health Research and development (NIHRD) (2018). Basic Health Research 2018 study guidelines and questionnaire (in Bahasa).

[12] WHO/FAO expert consultation (2003). Diet, nutrition and the prevention of chronic diseases : report of a joint WHO/FAO expert consultation, Geneva, 28 January - 1 February 2002.

[13] Armstrong T, Bull F (2006). Development of the World Health Organization global physical activity questionnaire (GPAQ). J Public Health (Bangkok).

[14] Beusenberg M, Orley JH (1994). A User’s guide to the self reporting questionnaire (SRQ).

[15] Ganihartono I (1996). Psychiatric morbidity among patients attending the Bangetayu community health Centre in Indonesia. Bul Penelit Kesehat.

[16] WHO/IASO/IOTF (2000). The Asia-Pacific perspective: redefining obesity and its treatment.

[17] World Health Organisation (WHO) (2011). Waist Circumference and Waist–Hip Ratio. Report of a WHO Expert Consultation.

[18] Browning LM, Hsieh SD, Ashwell M (2010). A systematic review of waist-to-height ratio as a screening tool for the prediction of cardiovascular disease and diabetes: 0.5 could be a suitable global boundary value. Nutr Res Rev.

[19] Chobanian AV, Bakris GL, Black HR, Cushman WC, Green LA, Izzo JL (2003). Seventh report of the joint National Committee on prevention, detection, evaluation, and treatment of high blood pressure. Hypertension..

[20] American Diabetes Association (2021). 2. Classification and Diagnosis of Diabetes: Standards of Medical Care in Diabetes—2021. Diabetes Care.

[21] Grundy SM, Stone NJ, Bailey AL, Beam C, Birtcher KK, Blumenthal RS (2019). 2018 AHA/ACC/AACVPR/AAPA/ABC/ACPM/ADA/AGS/APhA/ASPC/NLA/PCNA guideline on the Management of Blood Cholesterol: a report of the American College of Cardiology/American Heart Association task force on clinical practice guidelines. Circulation..

[22] Barros AJ, Hirakata VN (2003). Alternatives for logistic regression in cross-sectional studies: an empirical comparison of models that directly estimate the prevalence ratio. BMC Med Res Methodol.

[23] World Health Organization (WHO). The fatal link between tobacco and cardiovascular diseases in the WHO South-East Asia region: WHO south-east Asian regional Office; 2018.

[24] Reitsma MB, Kendrick PJ, Ababneh E, Abbafati C, Abbasi-Kangevari M, Abdoli A (2021). Spatial, temporal, and demographic patterns in prevalence of smoking tobacco use and attributable disease burden in 204 countries and territories, 1990–2019: a systematic analysis from the global burden of disease study 2019. Lancet..

[25] Yusuf S, Hawken S, Ôunpuu S, Dans T, Avezum A, Lanas F (2004). Effect of potentially modifiable risk factors associated with myocardial infarction in 52 countries (the INTERHEART study): case-control study. Lancet..

[26] National Institute of Health Research and Development (NIHRD). National Basic Health Research (Riskesdas) Report 2013. Jakarta; 2013. http://labdata.litbang.kemkes.go.id/images/download/laporan/RKD/2013/Laporan_riskesdas_2013_final.pdf. Accessed 15 Aug 2021.

[27] Ng M, Freeman MK, Fleming TD, Robinson M, Dwyer-Lindgren L, Thomson B (2014). Smoking prevalence and cigarette consumption in 187 countries, 1980-2012. JAMA..

[28] Afshin A, Sur PJ, Fay KA, Cornaby L, Ferrara G, Salama JS (2019). Health effects of dietary risks in 195 countries, 1990–2017: a systematic analysis for the global burden of disease study 2017. Lancet..

[29] Kalmpourtzidou A, Eilander A, Talsma EF (2020). Global vegetable intake and supply compared to recommendations: a systematic review. Nutrients..

[30] Miller V, Mente A, Dehghan M, Rangarajan S, Zhang X, Swaminathan S (2017). Fruit, vegetable, and legume intake, and cardiovascular disease and deaths in 18 countries (PURE): a prospective cohort study. Lancet..

[31] Wahid A, Manek N, Nichols M, Kelly P, Foster C, Webster P, et al. Quantifying the association between physical activity and cardiovascular disease and diabetes: a systematic review and meta-analysis. J Am Heart Assoc. 2016;5:e002495 10.1161/JAHA.115.002495 PMC5079002 27628572

[32] Guthold R, Stevens GA, Riley LM, Bull FC (2018). Worldwide trends in insufficient physical activity from 2001 to 2016: a pooled analysis of 358 population-based surveys with 1·9 million participants. Lancet Glob Health.

[33] Doyle F, McGee H, Conroy R, Conradi HJ, Meijer A, Steeds R (2015). Systematic review and individual patient data Meta-analysis of sex differences in depression and prognosis in persons with myocardial infarction. Psychosom Med.

[34] Gale CR, Batty GD, Osborn DPJ, Tynelius P, Rasmussen F (2014). Mental disorders across the adult life course and future coronary heart disease. Circulation..

[35] Nur KRM (2018). Illness perception and cardiovascular health behaviour among persons with ischemic heart disease in Indonesia. Int J Nurs Sci.

[36] Rahman ARA, Wang J-G, Kwong GMY, Morales DD, Sritara P, Sukmawan R (2015). Perception of hypertension management by patients and doctors in Asia: potential to improve blood pressure control. Asia Pac Fam Med.

[37] Webster R, Heeley E (2010). Perceptions of risk: understanding cardiovascular disease. Risk Manag Healthc Policy.

[38] Liu J, Tse LA, Liu Z, Rangarajan S, Hu B, Yin L (2019). Predictive values of anthropometric measurements for Cardiometabolic risk factors and cardiovascular diseases among 44 048 Chinese. J Am Heart Assoc.

[39] Agbo HA, Zoakah AI, Isichei CO, Sagay AS, Achenbach CJ, Okeahialam BN. Cardiovascular anthropometry: what is best suited for large-scale population screening in sub-Saharan Africa? Front Cardiovasc Med. 2020;7:1–7. 10.3389/fcvm.2020.522123 PMC7744454 33344511

[40] Hussain MA, Al Mamun A, Peters SA, Woodward M, Huxley RR (2016). The burden of cardiovascular disease attributable to major modifiable risk factors in Indonesia. J Epidemiol.

[41] Ahmad N, Adam SM, Nawi A, Hassan M, Ghazi H (2016). Abdominal obesity indicators: waist circumference or waist-to-hip ratio in Malaysian adults population. Int J Prev Med.

[42] Truthmann J, Busch MA, Scheidt-Nave C, Mensink GBM, Gößwald A, Endres M (2015). Modifiable cardiovascular risk factors in adults aged 40–79 years in Germany with and without prior coronary heart disease or stroke. BMC Public Health.

[43] Karastergiou K, Smith SR, Greenberg AS, Fried SK (2012). Sex differences in human adipose tissues – the biology of pear shape. Biol Sex Differ.

[44] Chan YY, Sahril N, Rezali MS, Kuang Kuay L, Baharudin A, Abd Razak MA (2021). Self-reported modifiable risk factors of cardiovascular disease among older adults in Malaysia: a cross-sectional study of prevalence and clustering. Int J Environ Res Public Health.

[45] Gu D, Gupta A, Muntner P, Hu S, Duan X, Chen J (2005). Prevalence of cardiovascular disease risk factor clustering among the adult population of China. Circulation..

[46] Ministry of Health Republic of Indonesia (2019). Buku Pedoman Manajemen Penyakit Tidak Menular (Non Communicable Disease Management Guideline).

[47] Maharani A, Tampubolon G (2014). Unmet needs for cardiovascular Care in Indonesia. PLoS One.

[48] Mboi N, Murty Surbakti I, Trihandini I, Elyazar I, Houston Smith K, Bahjuri Ali P (2018). On the road to universal health care in Indonesia, 1990–2016: a systematic analysis for the global burden of disease study 2016. Lancet..

[49] Mosca L, Barrett-Connor E, Kass WN (2011). Sex/gender differences in cardiovascular disease prevention. Circulation..

